# Innovative dentifrices based on bioactive silica for enamel remineralization and erosion control: an in vitro study

**DOI:** 10.1186/s12903-025-07502-0

**Published:** 2025-12-09

**Authors:** Elizabeth Barreto Galvão Sousa, Anderson Gomes Forte, Juliellen Luiz Cunha, Marcel Alves Avelino Paiva, Vitória Régia Rolim Nunes, Adriana Moreira Ferreira, Ana Maria Barros Chaves Pereira, Fábio Correia Sampaio, Andressa Feitosa Bezerra Oliveira

**Affiliations:** 1 Graduate Program in Dentistry, Federal University of Paraíba (UFPB), João Pessoa, Brazil; 2 Graduate Program in Dental Materials, University of Campinas (UNICAMP), Piracicaba, Brazil; 3 School of Dentistry, Institutional Scientific Initiation Scholarship Program, Federal University of Paraíba (UFPB), João Pessoa, Brazil; 4 Department of Morphology, Federal University of Paraíba (UFPB), João Pessoa, Brazil; 5 Department of Clinical and Social Dentistry, Federal University of Paraíba (UFPB), João Pessoa, Brazil; 6 Federal University of Paraíba – Campus I, Health Sciences Center (CCS), Jardim Universitário, s/n, Castelo Branco, João Pessoa, PB 58051-900 Brazil

**Keywords:** Dental enamel, Toothpaste, Fluoride, Silica, Tooth erosion

## Abstract

**Background:**

Dental erosion and abrasion lead to progressive enamel loss, and preventive strategies often rely on fluoride dentifrices. Bioactive silica has emerged as a promising component due to its remineralizing potential, acting alone or synergistically with fluoride. This in vitro study evaluated the efficacy of bioactive silica dentifrices, with or without different fluoride concentrations, in promoting enamel remineralization and protecting against erosion‒abrasion cycles.

**Methods:**

Sixty bovine enamel samples were assigned to five groups: RGS1 (bioactive silica dentifrice with 1450 ppm F), RGS2 (bioactive silica dentifrice with 1100 ppm F), RGS3 (bioactive silica dentifrice without fluoride), positive control (PC, 1100 ppm F dentifrice), and negative control (NC, fluoride- and silica-free dentifrice). The samples were subjected to a 5-day erosion-abrasion cycle with daily treatment with the assigned dentifrices. Enamel changes were assessed through surface microhardness recovery (%SMHR), fluorescence recovery (%ΔFR), surface loss (ΔSL), and surface roughness variation (ΔRa). The data were analyzed via one-way and repeated-measures ANOVA, followed by Tukey’s post hoc test (α = 0.05).

**Results:**

All tested dentifrices significantly affected enamel properties. Compared with the controls, bioactive silica dentifrices improved the microhardness and promoted greater mineral gain (*p* < 0.05). RGS2 demonstrated the highest %SMHR and ΔFR values, along with lowest surface roughness and enamel loss, indicating superior protective potential. RGS1, RGS3 and PC followed, with intermediate outcomes. The NC group exhibited the poorest performance across all the parameters evaluated (*p* < 0.05).

**Conclusion:**

Bioactive silica dentifrices significantly enhance enamel remineralization and protection against erosion-abrasion. The results highlight both the synergistic and independent roles of bioactive silica, supporting its application in daily oral care strategies for managing enamel wear.

**Clinical trial number:**

Not applicable.

## Background

Dental erosion is a noncarious dental lesion characterized by the progressive loss of mineral content from the tooth surface due to acid exposure, in the absence of bacterial involvement [1, 2]. Etiological factors can be classified as extrinsic, such as frequent consumption of acidic foods, beverages, or medications, and intrinsic, including conditions such as gastroesophageal reflux or eating disorders that expose teeth to gastric acids [3, 4]. Acid-induced demineralization softens the enamel, increasing its susceptibility to mechanical wear, especially from toothbrushing [2]. Erosive tooth wear (ETW) is now recognized as a multifactorial condition, in which chemical, mechanical, and behavioral factors interact to accelerate the degradation of hard dental tissues [2, 5]. If not diagnosed and managed in its early stages, ETW may lead to dentin exposure, hypersensitivity, loss of the occlusal vertical dimension, and compromised aesthetics [6, 7]. To mitigate ETW, fluoride-based strategies have been widely adopted. Monovalent fluoride, such as sodium fluoride (NaF), promotes the formation of a calcium fluoride-like (CaF₂) layer on the enamel surface [8, 9]. This transient protective layer acts as a fluoride reservoir, releasing ions under acidic conditions [9, 10]. However, this layer, although relatively insoluble at neutral pH, can be partially removed by brushing and salivary flow, particularly under erosive conditions [2, 8, 11].

In response to these limitations, novel dentifrice formulations have been developed following the principles of preventive and minimally invasive dentistry, aiming to increase enamel protection and promote remineralization [12, 13]. Among these advancements, bioactive silica (SiO₂-based compounds) has emerged as a promising agent capable of enhancing remineralization dynamics [14]. These compounds facilitate the deposition of calcium and phosphate ions and support the formation of a new mineralized layer on demineralized enamel, thereby improving its resistance to acid challenges [14]. In clinical scenarios that require enamel repair, bioactive agents capable of inducing hydroxyapatite nucleation are particularly valuable owing to their increased integration with dental structures [14–16].

Notably, the combination of bioactive silica with fluoride may result in synergistic effects, resulting in improved surface stability and greater resistance to acid challenges [17]. Previous studies have investigated the combined effects of bioactive silica, sodium fluoride, and tetrasodium pyrophosphate on the recovery of eroded enamel via pH cycling models [11, 17, 18]. These investigations revealed that dentifrice formulations containing both bioactive silica and fluoride, particularly in acidified environments, were more effective than fluoride alone in restoring enamel surface hardness and promoting structural repair. Such formulations were specifically designed to stimulate the formation of fluoride-enriched hydroxyapatite layers, often described as “enamel-like” structures [11]. These biomimetic layers exhibit enhanced resistance to acid dissolution and promote subsurface enamel remineralization, typically within the first 10–30 μm of the surface, as demonstrated by previous QLF and microradiographic analyses [17–20].

Based on these premises, this in vitro study aimed to evaluate the efficacy of dentifrices containing bioactive silica, with or without fluoride at different concentrations, in repairing enamel subjected to erosive–abrasive cycling. The null hypothesis tested was that the incorporation of bioactive silica, regardless of fluoride presence or concentration, would not result in statistically significant differences in (1) enamel surface hardness recovery, (2) post treatment mineral uptake, or (3) reduction in enamel surface loss compared with fluoride- and silica-free placebo dentifrice.

## Methods

### Sample preparation

 Freshly extracted bovine incisors were stored in a 0.08% thymol solution until use. These teeth were obtained from a certified slaughterhouse as post-mortem by-products, and no animals were sacrificed for research purposes. The use of animal-derived tissues followed institutional guidelines and international ethical principles. The sample size calculation was based on a previous study [21] that reported a large effect size (Cohen’s d = 1.29), with parameters set to achieve 80% statistical power and a 5% significance level, yielding a minimum of nine samples per group. To increase statistical confidence and compensate for potential specimen loss, 12 enamel blocks were prepared for each group. Samples were randomly assigned to the experimental groups by simple random allocation performed by an independent researcher, ensuring that each specimen had an equal chance of being included in any group.

Standardized enamel blocks (4 × 4 × 2 mm) were obtained from the middle third of the buccal surface of bovine incisors to ensure uniformity of mineral content and surface hardness. The enamel slabs were then embedded in acrylic resin, and polished with silicon carbide papers (grit 600 to 1500) under continuous water irrigation. Final polishing was performed with a 1 μm diamond paste. The baseline surface microhardness (SH₀) was measured via a Vickers microhardness tester (Shimadzu HMV—AD Easy Test, Version 3.0) with five indentations per sample (50 g load, 10 s dwell time, 100 μm spacing), as described in previous studies [11, 17, 18], allowing reproducible comparisons with similar experimental models. Only the samples with initial hardness values of 380 ± 10 VHN were included.

**Fig. 1:**
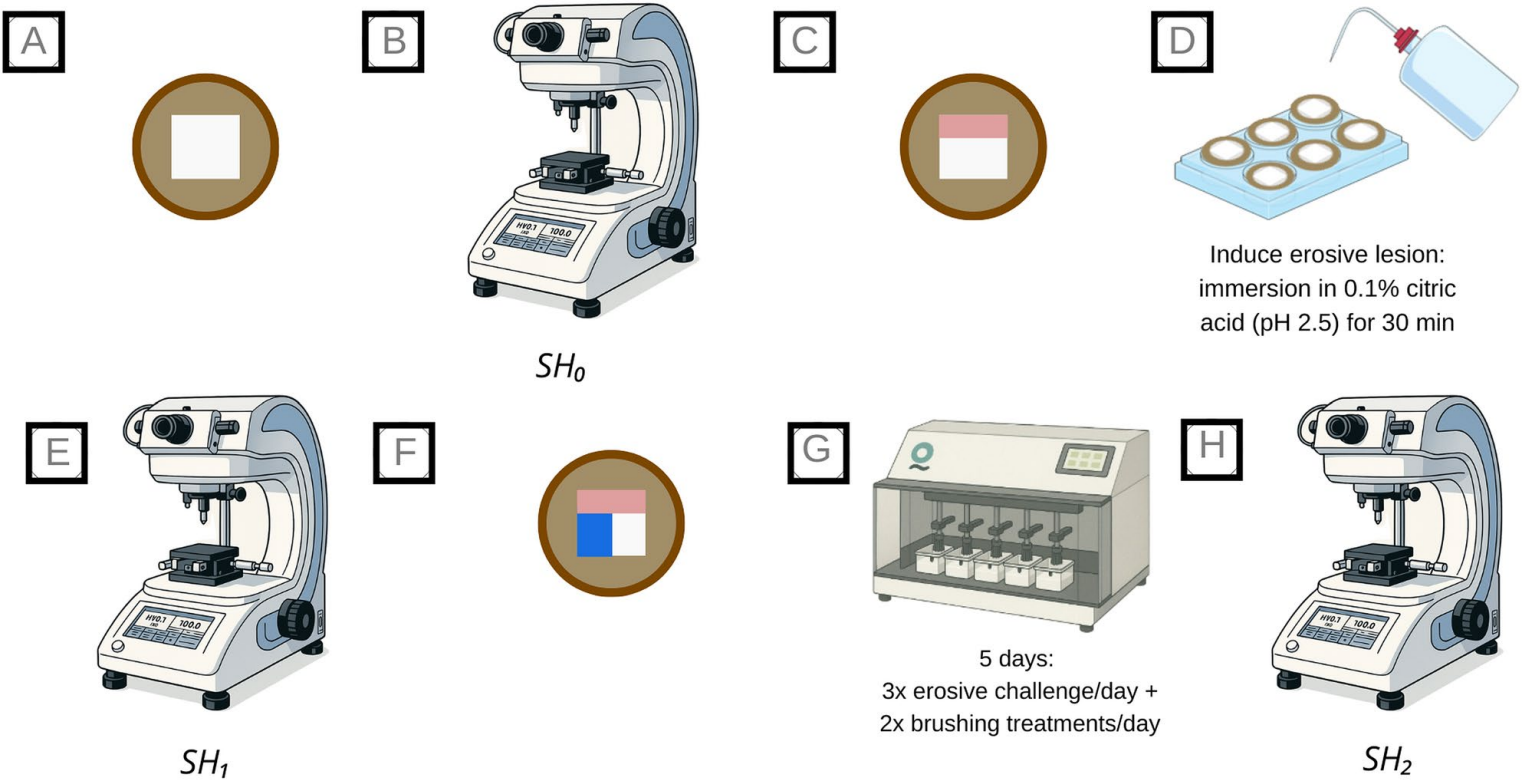
Experimental workflow of the erosive–abrasive model used in this study. **A** Enamel blocks embedded in acrylic resin and polished. **B** Baseline surface microhardness (SH₀). **C** Protection of the sound reference area with acid-resistant nail varnish. **D** Induction of the erosive lesion: immersion in 0.1% citric acid (pH 2.5) for 30 min under gentle agitation. **E** Post-erosion surface microhardness (SH₁). **F** Protection of the eroded area to delimit the treatment window. **G** Five-day erosion–abrasion cycling at 37 °C: 3x daily erosive challenge (90 s each) + 2x daily brushing treatments (15 s + 45 s immersion in dentifrice slurry). (H) Post-treatment surface microhardness (SH₂)

Each enamel block was divided into three distinct regions: (1) a sound control area, protected before lesion formation; (2) an eroded control area, created by acid exposure and covered after erosion; and (3) a treatment area, corresponding to the eroded enamel exposed to the dentifrice. To protect regions 1 and 2 (Fig. 1), an acid-resistant nail varnish (Risqué^®^, Niasi, Taboão da Serra, SP, Brazil) was applied in two thin layers. The first coat was allowed to dry completely before applying the second, using a freshly opened bottle to ensure optimal adhesion. This procedure effectively prevented varnish detachment during brushing, and no sample loss occurred throughout the experimental protocol.

### Lesion formation

Enamel erosion lesions were induced by immersing the specimens in a 0.3% citric acid solution (pH 2.5) for 30 min at room temperature (28 °C) under continuous agitation (60 rpm) [22]. To maintain a consistent erosive potential, the acid solution (30 mL per specimen) was refreshed every 5 min. This pre-erosion was designed to produce standardized lesions of clinically relevant depth, consistent with previous in vitro studies on bovine enamel under controlled erosive conditions [11, 21–23]. Following lesion formation, surface microhardness was reassessed in the eroded region (SH₁) using the same parameters described previously [18].

### Experimental groups and slurry preparation

Five dentifrices were tested, as described in Table 1. Products were coded and stored by an independent researcher to maintain blinding during treatment and data analysis. Dentifrice slurries were freshly prepared daily by mixing each formulation with deionized water at a 1:3 ratio (w/w) and homogenized under constant agitation for 4 min prior to use.

**Table 1:** Composition and manufacturer information of the dentifrice formulations evaluated in this study

Products (Groups)	Active Ingredients*	Manufacturer
Sensitive Regenerator (RGS 1)	Si-dentifrice with 1450 ppm sodium fluoride (REFIX^®^ Technology).	Rabbit Corp, Londrina, PR, Brasil (Lot number: 73045)
Sensitive Regenerator (RGS 2)	Si-dentifrice with 1110 ppm sodium fluoride (REFIX^®^ Technology).	Rabbit Corp, Londrina, PR, Brasil (Lot number: 020/2023)
Sensitive Regenerator (RGS 3)	Si-dentifrice with no Fluoride (REFIX^®^ Technology).	Rabbit Corp, Londrina, PR, Brasil (Lot number: 083/2023)
Negative Control (NC)	Fluoride-free dentifrice	Rabbit Corp, Londrina, PR, Brasil (Lot number: 74071)
Positive Control (PC)	1100 ppm sodium fluoride	Rabbit Corp, Londrina, PR, Brasil (Lot number: 07802021)

*Manufacturer information: all products provided by Rabbit Corp, Londrina, PR, Brazil

### pH cycling and erosion-abrasion protocol

Prior to the initiation of pH cycling, the samples were immersed in a remineralizing solution for 24 h. The experimental model then simulated daily erosive and abrasive challenges over a 5-day period at 37 °C, following a protocol adapted from Simões et al. (2020) [21]. Each day, before and after the erosive challenge, the samples were stored in artificial saliva (0.2 mM glucose, 9.9 mM NaCl, 1.5 mM CaCl₂·2 H₂O, 3 mM NH₄Cl, 17 mM KCl, 2 mM NaSCN, 2.4 mM K₂HPO₄, 3.3 mM urea, 2.4 mM NaH₂PO₄, and 11 µM ascorbic acid, pH 6.8), as described by Magalhães et al. (2008) [24]. The erosive challenge consisted of immersing each sample in 0.1% citric acid (pH 2.5) for 90 s three times per day under gentle agitation [21, 22]. After each cycle, the samples were rinsed with deionized water (10 s) and stored in artificial saliva (30 mL/specimen, pH 6.8, 25 °C) for two hours between challenges.

After the first and last erosive challenges of each day, abrasion was performed using an automated brushing machine (MEV 3T-8XY, Odeme, Joaçaba, Brazil). Soft-bristled toothbrush heads (Oral-B Indicator^®^, Procter & Gamble, São Paulo, Brazil) were used. Each session consisted of 10 s of active brushing with 30 mL of dentifrice slurry, followed by 110 s of passive exposure, totaling 2 min of treatment. The brushing motion comprised 11 vertical zigzag strokes (20 mm amplitude) performed at 37 °C under a constant 150 g load. After each day’s final cycle, the specimens were stored overnight in artificial saliva.

### Surface microhardness measurement

Surface microhardness was evaluated at three time points: baseline (SH₀), after erosion (SH₁), and after treatment (SH₂), using a Vickers indenter with a 50 g load and a 10-second dwell time. Five evenly spaced indentations (100 μm apart) were made per specimen. The percentage of surface microhardness recovery (%SMHR) was calculated as follows:

$$\%\mathrm{SMHR}=100\times\frac{\left(\mathrm{SH}2-\mathrm{SH}1\right)}{\left(\mathrm{SH}0-\mathrm{SH}1\right)}18$$

### Quantitative light-induced fluorescence (QLF) analysis

Mineral changes were assessed via quantitative light-induced fluorescence (QLF) imaging (Qraycam Pro, Inspektor Research Systems BV, Amsterdam, Netherlands). Prior to image acquisition, the nail varnish was carefully removed via acetone-soaked cotton swabs, followed by rinsing with deionized water and air drying.

After completing the pH-cycling protocol, quantitative light-induced fluorescence (QLF) images were acquired using a standardized setup to ensure consistent positioning and illumination conditions. All images were captured in a dark room, with exposure and contrast set to zero, and the device positioned at a fixed distance of 8 cm from the specimen. Each sample was placed in a custom acrylic holder to guarantee identical alignment across captures. The fluorescence data were analyzed with Q-ray software (version 1.38, Inspektor Research Systems BV, Amsterdam, Netherlands), which quantifies mineral changes based on fluorescence loss (ΔF). The control, eroded, and treated regions were evaluated within the same image, ensuring consistent measurement across all areas.

Two measurement stages were analyzed: ΔF₀, representing fluorescence loss between sound and eroded enamel, and ΔF₁, representing fluorescence loss between sound enamel and eroded enamel after dentifrice treatment. Fluorescence recovery (%ΔFR) was calculated as %ΔFR = [(ΔF₀ − ΔF₁)/ΔF₀] × 100, where positive values indicate mineral gain and negative values represent continued mineral loss [11].

### Surface profilometry analysis

Surface topography was assessed using a noncontact 3D optical profilometer (Talysurf CCI MP, Leicester, UK) operating under the following conditions: 20× magnification, 0.86 × 0.86 mm² field of view, XY reading mode with 1024 × 1024 px resolution, low-reflectance roughness setting (level 4), a 0.25 mm cutoff, and a standard Gaussian filter (ISO 16610-61).

Surface roughness (Ra) was measured at three time points: Ra₀ (sound enamel), Ra₁ (after erosion), and Ra₂ (after treatment). The change in roughness (ΔRa) was calculated as ΔRa = Ra₂ − Ra₁, where negative values indicate surface smoothing after treatment. Surface loss (SL) was determined from vertical step differences between adjacent regions: SL₀ (sound vs. eroded) and SL₁ (sound vs. treated). The variation in surface loss (ΔSL) was calculated as ΔSL = SL₁ − SL₀, where higher values correspond to greater enamel loss.

For each specimen, measurements were averaged from three predefined horizontal lines located at 25%, 50%, and 75% of the surface area. Three-dimensional color-coded surface maps were generated for qualitative visualization of enamel wear patterns.

### Statistical analysis

Data were analyzed using SPSS software (version 21.0, SPSS Inc., Chicago, IL, USA). The Shapiro–Wilk and Levene tests were applied to verify data normality and homogeneity of variances. As both assumptions were satisfied, no data transformation was required.

Intergroup differences in SH, %SMHR, ΔF, and SL were analyzed using one-way ANOVA followed by Tukey’s post hoc test. Intragroup comparisons (SH₀–SH₂ and Ra₀–Ra₂) were performed using repeated-measures ANOVA after verifying sphericity. Statistical significance was set at α = 0.05.

## Results

### Surface microhardness

Table 2 shows the mean and standard deviation values of enamel surface microhardness at baseline (SH₀), after erosion (SH₁), and after treatment (SH₂) for each experimental group. No significant differences were observed for SH₀ or SH₁ (ANOVA, *p* > 0.05), confirming baseline homogeneity and consistent lesion formation across all groups. Repeated-measures ANOVA confirmed a significant decrease in microhardness after erosion (SH₀ vs. SH₁, *p* < 0.001) and partial recovery after treatment (SH₁ vs. SH₂, *p* < 0.001), demonstrating effective demineralization followed by remineralization.

After treatment (SH₂), significant differences were found among groups (one-way ANOVA, *p* < 0.001), except between RGS1 and PC (*p* > 0.05). The RGS2 group (bioactive silica + 1100 ppm fluoride) showed the highest surface microhardness recovery (%SMHR), approximately 23% greater than that of the positive control (*p* < 0.05). Among the REFIX^®^-containing formulations, %SMHR followed the trend RGS2 > RGS1 > RGS3. No significant differences were observed between PC and either RGS1 or RGS3 (*p* > 0.05), whereas the negative control (NC) exhibited the lowest %SMHR values.

**Table 2:** Mean (± SD) enamel surface microhardness at baseline (SH₀), after erosion (SH₁), after treatment (SH₂), and recovery (%SMHR) across experimental groups*

Group	SH 0	SH 1	SH 2	%SMHR
RGS 1	388.2 (6.4) a	190.3 (3.8) a	308.9 (5.4) b	59.9 (2.6) b
RGS 2	390 (6.6) a	193.4 (7.3) a	334.2 (13.2) c	71.7 (6.0) c
RGS 3	387.6 (5.2) a	190.7 (6.7) a	291.5 (17.9) d	51.2 (9.3) d
NC	391.2 (7.8) a	193 (7.4) a	210.9 (14) a	8.9 (8.9) a
PC	389.6 (7.8) a	192 (6.5) a	306.8 (10.3) b	58.1 (5.7) b, d

Groups sharing the same letter are not significantly different

*Lowercase superscript letters (a, b, c, d) indicate statistically significant differences between groups within the same time point (one-way ANOVA with Tukey’s post hoc, *p* < 0.05)

### Quantitative light-induced fluorescence (QLF) analysis

Table 3 summarizes the mean fluorescence loss values before and after treatment. No significant differences were observed among groups at ΔF₀ (*p* > 0.05), confirming comparable lesion severity before treatment. After treatment (ΔF₁), however, significant differences emerged (ANOVA, *p* < 0.001). Specimens treated with bioactive silica dentifrices (RGS1, RGS2, RGS3) exhibited substantially greater fluorescence recovery (~ 52%) than the positive control (~ 15%) and the negative control, which showed continued mineral loss. No significant differences were detected among the three bioactive silica formulations (*p* > 0.05).

The percentage of fluorescence recovery (%ΔFR) followed the same trend, with the bioactive silica groups presenting the highest values (*p* < 0.05), whereas the negative control was the only group with a negative %ΔFR, indicating ongoing demineralization.

**Table 3:** Mean (± SD) quantitative light-induced fluorescence values before (ΔF₀) and after treatment (ΔF₁), and fluorescence recovery (%ΔFR)*

Group	ΔF 0	ΔF 1	%ΔFR
RGS1	−12.6 (1.0) a	−6.5 (0.5) c	47.9 (5.9) c
RGS2	−12.7 (1.0) a	−6.7 (0.6) c	46.5 (6.5) c
RGS3	−13.0 (0.8) a	−6.6 (0.5) c	48.6 (5.1) c
NC	−12.8 (1.0) a	−14.7 (1.8) a,	−13.0 (3.1) a
PC	−12.5 (0.8) a	−10.6 (0.7) b	14.7 (2.9) b

Groups sharing the same letter are not significantly different

*Lowercase superscript letters (a, b, c) indicate statistically significant differences between groups within the same time point (one-way ANOVA with Tukey’s post hoc, *p* < 0.05)

### Surface roughness and topography

**Fig. 2:**
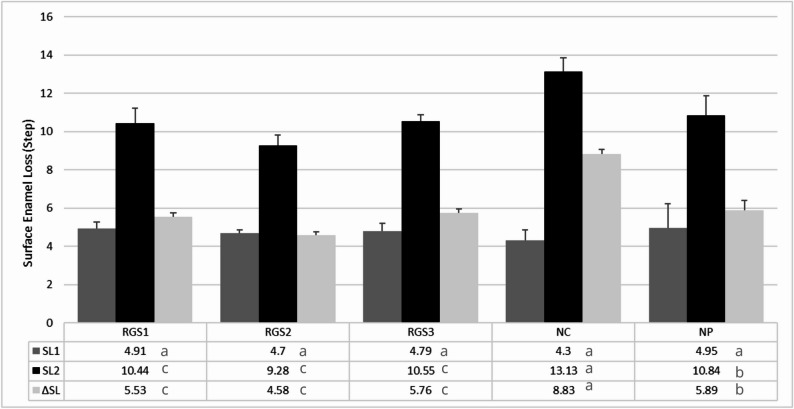
Enamel Surface loss (step height, µm) of bovine enamel across the experimental groups, expressed as mean values for SL₁ (after erosion), SL₂ (after treatment), and ΔSL (change in surface loss). Lowercase superscript letters (a, b, c) indicate statistically significant differences between groups (one-way ANOVA with Tukey’s post hoc, *p* < 0.05)

**Fig. 3:**
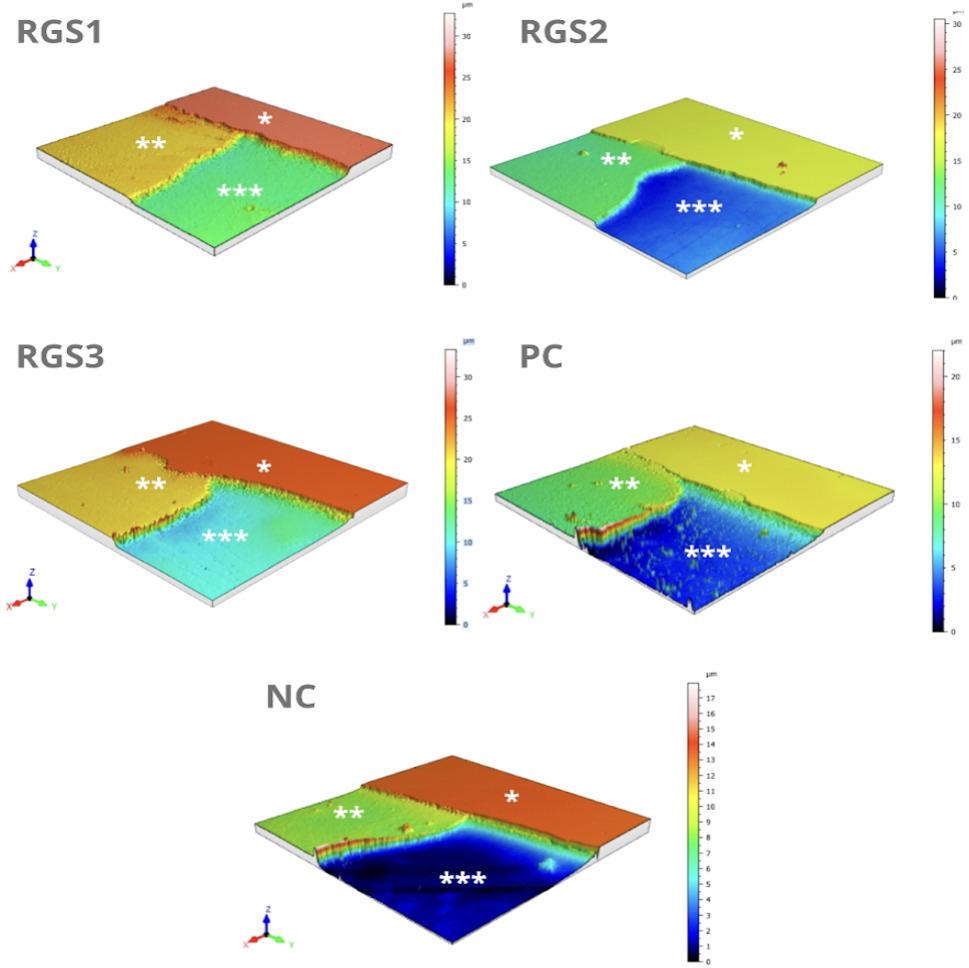
Representative three-dimensional optical profilometry images of enamel surfaces from the experimental groups. Each panel shows three regions: (*) sound enamel; (**) eroded enamel; and (***) treated enamel surface. Color maps represent surface height variations (orange = least surface loss; dark blue = greatest loss) Below Fig. 3: Vertical scales (30 μm and 17 μm) were adjusted only to optimize visualization of topographic features; all quantitative comparisons were based on profilometric ΔSL measurements

Table 4; Figs. 2 and 3 present the quantitative and qualitative profilometric analyses of enamel surface changes. No significant differences were found for Ra₀ or Ra₁ among the groups (*p* > 0.05), confirming consistent baseline conditions and standardized lesion formation. Repeated-measures ANOVA indicated significant changes within all groups (*p* < 0.05), with Ra₁ values higher than Ra₀ and partial recovery after treatment (Ra₂ < Ra₁).

After treatment, the negative control exhibited the highest roughness (Ra₂ = 0.282 μm), significantly higher than that of RGS2 (*p* < 0.05). The positive control showed intermediate roughness (0.210 μm), while RGS1 and RGS3 displayed similar values, with no significant differences from PC or RGS2. Regarding ΔRa, all bioactive silica groups (RGS1, RGS2, RGS3) showed negative values, indicating surface smoothing after treatment. RGS2 achieved the most pronounced effect (ΔRa = − 0.149 μm), significantly superior to PC (–0.055 μm) and NC (+ 0.056 μm) (*p* < 0.05). The positive ΔRa observed for NC reflects a deterioration in surface texture following treatment.

**Table 4:** Mean (± SD) enamel surface roughness at baseline (Ra₀, sound enamel), after erosion (Ra₁), and after treatment (Ra₂), and roughness change (ΔRa, µm) across experimental groups*

Group	Ra 0	Ra 1	Ra 2	ΔRa
RGS1	0.034 (0.022) a	0.234 (0.064) a	0.159 (0.037) b, c	–0.075 (0.033) b
RGS2	0.019 (0.008) a	0.275 (0.068) a	0.126 (0.044) c	–0.149 (0.024) c
RGS3	0.029 (0.017) a	0.254 (0.043) a	0.150 (0.045) b, c	–0.104 (0.032) c
NC	0.019 (0.009) a	0.226 (0.069) a	0.282 (0.062) a	+ 0.056 (0.046) a
PC	0.021 (0.006) a	0.265 (0.061) a	0.210 (0.055) b	−0.055 (0.041) b

Groups sharing the same letter are not significantly different

* Lowercase superscript letters (a, b, c) indicate statistically significant differences between groups within the same time point (one-way ANOVA with Tukey’s post hoc, *p* < 0.05)

Surface loss (SL) followed a consistent trend. SL₀ values (sound vs. eroded) did not differ among groups (*p* > 0.05), confirming standardized lesion depth (Fig. 2). After treatment (SL₁), the NC group exhibited the greatest surface loss (*p* < 0.05), whereas RGS2 presented the lowest values. RGS1, RGS3, and PC achieved intermediate performance, with no significant differences among them. All bioactive silica formulations (RGS1, RGS2, RGS3) demonstrated greater enamel preservation (lower ΔSL) compared with PC and NC.

Three-dimensional optical profilometry (Fig. 3) revealed distinct surface topographies across groups. Color-coded height maps illustrate preserved areas in orange/yellow, mild wear in green, and greater tissue loss in dark blue. The RGS2 group (bioactive silica + 1100 ppm F⁻) exhibited the smoothest and most uniform surface, dominated by green and light-blue regions, indicating minimal wear and well-preserved contours. RGS1 displayed a wider range of colors, from orange and yellow in intact areas to light-blue patches in slightly abraded zones, suggesting moderate localized wear. RGS3, despite being fluoride-free, presented a similar but slightly more worn surface, with larger blue regions.

In contrast, the NC group revealed extensive dark-blue areas, consistent with severe structural loss and irregular morphology, while the PC group showed mainly light-blue surfaces, indicating moderate erosion. Compared with PC, RGS2 maintained a smoother and more continuous enamel profile, confirming the superior protective effect of the bioactive silica formulation containing 1100 ppm fluoride.

## Discussion

The results of this study support the rejection of all three null hypotheses, as the incorporation of bioactive silica, particularly when combined with fluoride, significantly improved enamel surface properties. These findings highlight the multifactorial benefits of bioactive silica dentifrices in enhancing microhardness, reducing mineral loss, and promoting smoother surfaces, all of which are critical to mitigating erosive–abrasive wear [18, 19, 25, 26]. Taken together, these findings indicate that bioactive silica functions not as a conventional abrasive but as a bioactive agent capable of promoting enamel microstructural repair through ion release and apatite nucleation [11, 14, 16].

Among the tested formulations, RGS2 (1100 ppm F⁻) achieved the highest %SMHR and %ΔFR values and the lowest ΔSL and ΔRa measurements, indicating superior enamel recovery and surface preservation. This suggests that the optimized fluoride concentration favored the formation of a cohesive and uniform mineral layer, improving surface hardness and resistance to wear [11, 12, 25, 27, 28].

Although RGS1 (1450 ppm F⁻) contained the same bioactive matrix, its higher fluoride content did not produce proportional benefits. This may be attributed to physicochemical interactions between fluoride and silica, in which excessive fluoride accelerates the precipitation of calcium-fluoride-like (CaF₂) deposits, forming a dense and less reactive surface layer [9, 11, 12]. Such a barrier restricts ion diffusion and limits silica–enamel interaction, hindering homogeneous fluorapatite formation and favoring superficial rather than subsurface mineralization [11, 12, 22, 24, 29]. Consequently, an intermediate fluoride concentration, as in RGS2, provides more favorable conditions for enamel repair by promoting deeper ion exchange and more stable remineralization. These results emphasize the importance of formulation balance in dentifrice design, where ion synergy is more relevant than fluoride concentration alone [12, 17, 24, 27, 28, 30].

While RGS2 exhibited greater microhardness recovery, no significant differences in fluorescence recovery (%ΔFR) were observed among the bioactive silica groups. This apparent divergence likely reflects the analytical depths of the methods used: microhardness primarily measures surface-level rehardening, whereas QLF detects subsurface mineral changes. Previous studies indicate that surface recovery can precede deeper remineralization, leading to higher hardness values without proportional fluorescence variation [8, 11, 23]. Thus, all bioactive formulations likely promoted comparable mineral gain within the lesion body, but RGS2 achieved superior surface reinforcement due to its optimized fluoride–silica interaction.

The fluoride-free formulation (RGS3) also produced significant improvements in %SMHR and %ΔFR compared with NC, confirming the fluoride-independent remineralizing capacity of bioactive silica [14, 15, 23]. Silicate ions can promote apatite nucleation and stabilize amorphous calcium phosphate, facilitating mineral deposition even in the absence of fluoride [14–16]. These findings expand the understanding of silica’s bioactivity, showing that it can independently induce mineral precipitation and act as a nucleation center, which is particularly relevant for fluoride-free formulations [11, 13, 14, 19].

The incorporation of bioactive silica represents a promising strategy to overcome the limitations of traditional fluoride-based approaches. Upon contact with saliva, bioactive silicates hydrate and gradually release calcium (Ca²⁺), phosphate (PO₄³⁻), fluoride (F⁻), and silicate (SiO₄⁴⁻) ions into the surrounding medium [12, 15, 31]. These ions diffuse into demineralized enamel and form a silicon- and fluoride-enriched hydroxyapatite layer—often referred to as fluorosilicate apatite [11, 14, 16, 32]. This biomimetic mineral coating bonds with the underlying enamel, improving structural integrity and resistance to acid dissolution [11–13]. Within this process, silicate ions serve as nucleation centers that guide crystal growth and stabilize amorphous calcium phosphate [12, 14, 16, 18, 32], ultimately forming a durable and biologically integrated protective layer [16, 18, 25, 33].

In contrast, fluoride alone promotes remineralization mainly through the formation of a superficial calcium fluoride-like (CaF₂) layer that acts as a transient ion reservoir under acidic conditions [9, 31, 34]. However, this layer can be rapidly removed by brushing or salivary flow, reducing its protective effect [35, 36]. Moreover, formulations containing free calcium and phosphate often show limited efficacy because premature ion interaction with fluoride hinders mineral deposition onto enamel [30, 37]. These limitations highlight the advantages of bioactive silica, which can modulate ion release and sustain apatite nucleation through both fluoride-dependent and fluoride-independent mechanisms [14–16]. At the molecular level, silicate ions (SiO₄⁴⁻) can replace phosphate groups at the B-sites of hydroxyapatite, forming a silicon-enriched apatite, whereas fluoride ions occupy the A-sites, generating fluorapatite [11, 32]. While fluorapatite enhances chemical stability and acid resistance, silicon promotes biological integration by facilitating localized calcium and phosphate deposition and improving crystal growth [14–16]. The complementary incorporation of silicon and fluoride explains the synergistic behavior observed in RGS2, where surface stability and subsurface remineralization occurred simultaneously [14, 16–18, 25]. This structural duality, fluoride strengthening the crystal lattice and silicon enhancing mineral connectivity, illustrates how controlled ion interactions can optimize both durability and biomimetic enamel repair.

When combined, bioactive silica and fluoride produce synergistic effects that enhance both surface and subsurface enamel integrity [17, 30]. Formulations containing these components, particularly under mildly acidic conditions, have demonstrated superior structural recovery, reduced mineral loss, and smoother surfaces compared with fluoride-only dentifrices [11, 18, 25, 30]. The resulting fluoride-enriched hydroxyapatite, often described as an “enamel-like” layer, exhibits greater acid resistance and structural cohesion, offering long-lasting protection [14, 17, 31, 33].

Although other bioactive systems, such as CPP-ACP and nano-hydroxyapatite (n-HA), also promote remineralization, their mechanisms differ fundamentally from that of bioactive silica. CPP-ACP and n-HA primarily act as external ion reservoirs, favoring surface mineral deposition [13, 19, 38, 39]. In contrast, bioactive silica releases silicate ions that initiate in situ nucleation of silicon- and fluoride-enriched apatite, forming a cohesive, acid-resistant, and biologically integrated mineral layer [14].

The performance of the positive control (fluoride-only) group further reinforces this distinction. Although fluoride enhanced surface hardness and morphology [10, 30], these effects were restricted to superficial regions, as fluorapatite formation offers limited penetration into deeper lesions [31, 37]. Consequently, fluoride-only dentifrices may be less effective against progressive mineral loss [10, 35]. In contrast, bioactive silica supports deeper mineral integration and more comprehensive repair, representing a shift from surface-protective to regenerative strategies in preventive oral care [11, 14, 17, 32].

Profilometric findings corroborated these results: RGS2 exhibited the lowest ΔSL and ΔRa values, indicating superior surface preservation and smoother morphology. This has clinical relevance, as reduced roughness minimizes plaque retention, bacterial adhesion, and susceptibility to erosive or abrasive challenges [26, 30]. RGS1 and PC showed intermediate outcomes, reinforcing that both fluoride concentration and bioactive silica contribute to surface protection and structural resilience. From a translational perspective, these results support bioactive silica-based dentifrices as a strategy to mitigate enamel wear in individuals frequently exposed to acids or mechanical stress, aligning with the minimally invasive approach of modern dentistry [11, 14, 27].

Overall, bioactive silica—alone or combined with fluoride—effectively promotes enamel repair and protection under erosive–abrasive conditions. The superior performance of RGS2 underscores the importance of optimized formulations that balance ion synergy and controlled release. This combination represents a new generation of multifunctional dentifrices addressing both preventive and therapeutic demands in managing enamel erosion and wear.

Despite the inherent limitations of an in vitro design—such as the absence of salivary flow, pellicle formation, and other biological variables—this model provides a controlled and reproducible environment for evaluating the physicochemical behavior of dentifrices [23, 27, 28]. The artificial saliva used contained only inorganic ions, preventing the formation of a protein-based acquired pellicle, which is known to enhance enamel protection in vivo. This limitation has been well documented, as salivary proteins modulate ion diffusion and play a crucial role in resistance to erosion [40]. Furthermore, even artificial or stored human saliva cannot fully replicate in situ conditions because of the lack of enzymatic activity and continuous salivary renewal [41]. Nevertheless, artificial saliva composed solely of inorganic ions remains a reliable and widely accepted model for mechanistic evaluation of erosive–abrasive processes [11, 21, 22, 25, 30]. In vitro protocols therefore continue to represent an essential first step for screening and optimizing new formulations before advancing to in situ or clinical validation [23, 28].

Given its strong performance and versatility, bioactive silica emerges as a clinically relevant technology for the prevention and restoration of early enamel erosion. Future in situ and clinical studies are encouraged to validate these results and to support the translation of bioactive silica-based dentifrices into evidence-based oral care products for daily use.

## Conclusions

This in vitro study demonstrated that dentifrices containing bioactive silica significantly enhanced enamel remineralization and surface recovery after erosion–abrasion cycles. The formulation combining bioactive silica with 1100 ppm fluoride (RGS2) showed the most favorable outcomes, indicating a synergistic interaction that optimized ion exchange and mineral integration. The fluoride-free bioactive silica dentifrice (RGS3) also promoted mineral gain, confirming the fluoride-independent remineralizing capacity of silica. In this context, the results of this study support both the development of combined bioactive silica–fluoride technologies, offering synergistic benefits and the formulation of effective fluoride-free dentifrices, aligned with contemporary demands for safe, effective, and more natural solutions for the prevention and early repair of erosive enamel lesions.

## Data Availability

The datasets generated and/or analyzed during the current study are available from the corresponding author on reasonable request.
